# Immune Reconstitution and Safe Metabolic Profile after the Switch to Bictegravir/Emtricitabine/Tenofovir Alafenamide Fumarate among Virologically Controlled PLWH: A 96 Week Update from the BICTEL Cohort

**DOI:** 10.3390/v15061222

**Published:** 2023-05-23

**Authors:** Alessandro Lazzaro, Diana Bianchini, Elio Gentilini Cacciola, Ivano Mezzaroma, Mario Falciano, Carolina Andreoni, Caterina Fimiani, Letizia Santinelli, Luca Maddaloni, Ginevra Bugani, Giancarlo Ceccarelli, Claudio Maria Mastroianni, Gabriella d’Ettorre

**Affiliations:** 1 Department of Public Health and Infectious Diseases, Sapienza University of Rome, Policlinico Umberto I of Rome, 00185 Rome, Italy; alessandro.lazzaro@uniroma1.it (A.L.); bianchini.1581807@studenti.uniroma1.it (D.B.); elio.gentilini@uniroma1.it (E.G.C.); mario.falciano@uniroma1.it (M.F.); andreonicarolina@tiscali.it (C.A.); c.fimiani@policlinicoumberto1.it (C.F.); letizia.santinelli@uniroma1.it (L.S.); luca.maddaloni@uniroma1.it (L.M.); ginevra.bugani@uniroma1.it (G.B.); giancarlo.ceccarelli@uniroma1.it (G.C.); claudio.mastroianni@uniroma1.it (C.M.M.); 2 Department of Translational and Precision Medicine, Sapienza University of Rome, AOU Policlinico Umberto I of Rome, 00185 Rome, Italy; ivano.mezzaroma@uniroma1.it

**Keywords:** bictegravir, BIC/FTC/TAF, switch, HIV, antiretroviral, real-life, safety, efficacy, tolerability, immune reconstitution

## Abstract

Background: Bictegravir/emtricitabine/tenofovir alafenamide fumarate (BIC/FTC/TAF) is a recommended once-daily single-tablet regimen for the treatment of people living with HIV (PLWH). We aimed to assess efficacy, safety, and tolerability of BIC/FTC/TAF among PLWH, with a specific focus on people older than 55 years. Methods: We recruited an observational retrospective real-life cohort, including all PLWH who underwent a therapeutic switch to BIC/FTC/TAF, independently from the previous treatment regimen (the BICTEL cohort). Longitudinal nonparametric analyses and linear models were built. Results: After 96 weeks of follow-up, 164 PLWH were included, with 106 older than 55. Both the intention-to-treat and the per-protocol analysis showed low rates of virologic failure, independent of the pre-switch anchor drug. At week 96, a significant increase in CD4^+^ T cell count and in CD4^+^/CD8^+^ ratio was observed, inversely correlated with baseline immune status. Fasting serum lipid profile, total body weight, BMI, and hepatic function were not affected by the switch, without new onset of metabolic syndrome or weight gain. Compared to baseline, we observed a renal function worsening which is worthy of further follow-up. Conclusion: BIC/FTC/TAF is an effective, safe, and well-tolerated switching strategy for PLWH, especially among those older than 55.

## 1. Introduction

The single-tablet regimen based on bictegravir/emtricitabine/tenofovir alafenamide fumarate (BIC/FTC/TAF) is a pharmaceutical option among the available antiretroviral drugs against the Human Immunodeficiency Virus type-1 (HIV-1) infection. It consists of a second-generation integrase strand transfer inhibitor (INSTI), namely bictegravir (BIC), and a tenofovir-based nucleos(t)ide backbone (FTC/TAF) [1,2,3]. It comes with some advantages, joining a second-generation INSTI that grants a high genetic barrier to resistance, which account for high virologic efficacy [4], and the pharmakocinetic properties of TAF, a prodrug of tenofovir, which allows restricted distribution of the active drug to those cells with high carboxyesterase and catepsin A, such as lymphocytes, minimizing the side effects associated with systemic exposure to other pharmaceutical forms of tenofovir (tubular nephropathy and osteoporosis) [5].

The pivotal randomized clinical trials of BIC/FTC/TAF showed it as a safe and effective therapeutic switching strategy for PLWH under virologic control, proving the remarkable properties of this once daily single-tablet regimen. Nonetheless, data from real-life experience are still lacking for time spans longer than 48 weeks [6,7,8] and for those PLWH usually excluded from pivotal studies, such as women, ethnical minorities, and the elderly [9].

In the current scenario, featured by highly active antiretroviral therapy (ART) (without virus eradication) and persistent chronic inflammation/immune activation, PLWH are experiencing a comparable lifespan to those without HIV-1 infection, but with early onset of comorbidities, usually observed in the elderly among the general population [10]. Aging and frailty are emerging as challenging issues impairing the management of the inflammaging sequelae in the daily out-patient setting. PLWH’s median age in high-income countries is about 55 years, with projections for the proportion of those over 65 to increase significantly in the next 10 years [11]. Nonetheless, older PLWH have rarely been studied systematically regarding ART efficacy, drug interactions, and tolerability [12].

Here, we show data of PLWH enrolled in the BICTEL cohort [13] after 96 weeks (w96) from the switch to BIC/FTC/TAF, with a specific focus on those older than 55. Moreover, we add interesting evidence of inverse correlation between the baseline (BL) immune status before the switch and the immune reconstitution observed at w96.

## 2. Materials and Methods

### 2.1. Study Design

The BICTEL cohort is an observational retrospective real-life cohort describing data from PLWH who switched their current ART to a single-tablet regimen based on BIC/FTC/TAF. It includes all PLWH who underwent a therapeutic switch to BIC/FTC/TAF, independently from the previous ART regimen.

The primary objective was to assess the virologic efficacy of BIC/FTC/TAF in a real-life cohort after 96 weeks of retrospective observation. The primary endpoint was the rate of virologic control (defined as HIV-RNA < 50 copies/mL) at week 96 (w96), defined using the US Food and Drug Administration (FDA)’s Snapshot algorithm [14]. Secondary objectives of the study were: (a) Immune changes from BL to w96 (secondary endpoint: CD4^+^ T cells, CD8^+^ T cells and CD4^+^/CD8^+^ ratio), (b) metabolic changes from BL to w96 (secondary endpoints: Fasting serum total (TC), LDL and HDL cholesterol, TC/HDL ratio, body weight, BMI, creatinine, estimated glomerular filtration rate (eGFR), (c) epidemiologic definition of the reason to switch to BIC/FTC/TAF (secondary endpoint: Information extracted from the medical records), (d) tolerability profile at w96 (secondary endpoints: Adverse events reported from BL to w96), (e) validation of the above mentioned primary and the secondary objectives among a sub-group of PLWH older than 55 years old, (f) validation of our previous observation at week 48 (w48) on larger sample size.

This study enrolled PLWH included in the BICTEL cohort with a follow-up of at least 96 weeks. The following information was extracted from the cohort database: Age, gender, smoking, time from HIV-1 diagnosis (years), previous AIDS diagnosis, hepatitis B virus (HBV) and hepatitis C virus (HCV) infection, presence of comorbidities (diabetes, hypertension, cardiovascular disease (CVD), chronic kidney disease (CKD), osteoporosis), number of non-ART co-medications, time with HIV-1 RNA < 50 copies/mL before switch (months), body weight (Kg), body mass index (BMI), creatinine (mg/dL), eGFR as measured by CKD-EPI formula (mL/min/1.73 m^2^) [15], TC, LDL, and HDL (mg/dL), TC/HDL ratio, HIV-RNA as copies/mL, CD4^+^ T lymphocytes (cells/µL), CD8^+^ T lymphocytes (cells/µL) and CD4^+^/CD8^+^ ratio, clinical assessment of side effects of the drug. Adverse events (AEs) were classified as mild/moderate, severe, or life-threatening, according to the Division of AIDS (DAIDS) Classification [16]. AEs were considered unrelated to BIC/FTC/TAF, possibly related or related, according to the physician’s evaluation.

Available information on historical pre-existing resistance-associated mutations (RAMs) nucleoside/nucleotide reverse transcriptase (NRTI), and integrase strand transfer inhibitors (INSTI) were collected in order to retrospectively observe the impact of unpredicted RAMs on the regimen efficacy. Participants with virologic failure (VF), as defined by HIV-1 RNA ≥ 200 copies/mL, underwent genotypic resistance test (GRT). RAMs were considered treatment-emergent when they were detected at GRT during the observation for those participants without historical genotypic data and when they were not previously present in historical data for participants with available GRT at BL.

### 2.2. Statistical Analysis

Since the BICTEL cohort keeps enrolling participants, thus increasing in sample size over time, the statistical analysis provides for a re-analysis of BL participants’ characteristics, as well as a re-analysis of the changes from BL to w48, together with the w96 versus BL and w96 versus w48 comparisons. BL characteristics of enrolled patients were considered as median values and interquartile range (IQR), or simple frequencies (#) and proportions (percentages, %), according to the variable type, continuous or categorical, respectively. Normality of variable distribution was assessed by the Shapiro–Wilk statistics. The Clopper–Pearson exact method was used to calculate 95% confidence intervals (CIs) for virologic data with both the intention-to-treat (missing as failure) and the per-protocol (missing as excluded) approaches. The change from BL was assessed by raw absolute difference (w96 value—BL value, w96 value—w48 value, w48 value—BL value) or percentage of median relative difference (MRD_(w96−BL)/BL_: 100 × [week 96 value—BL value]/BL value, MRD_(w96−48)/48_: 100 × [week 96 value − 48 value]/48 value, MRD_(w48−BL)/BL_: 100 × [week 48 value − BL value]/BL value). Longitudinal analysis was assessed by the paired Wilcoxon test for continuous variables and by Fisher’s exact test for categorical variables. Associations were assessed by univariable and multivariable linear regression models. All tests were two-sided, and a *p*-value of less than 0.05 was considered statistically significant. All data were analyzed using RStudio (Version 2022.12.0+353 Copyright© 2022 by Posit Software, PBC) and Microsoft Excel for Mac (Version 16.48).

### 2.3. Ethical Aspects

The study was independently approved by the Committee of the Public Health and Infectious Diseases Department of “Sapienza”, University of Rome and by the local Ethics Committee (No. of approval 0280/2021-6199, 31 March 2021). Written informed consent was obtained from each participant prior to the enrolment.

## 3. Results

### 3.1. Population Features

At the end of the enrolment, a total of 164 PLWH were included in the study. Compared to our previous report on the w48 follow-up [13], the current cohort has increased by 17 participants, from 147 to 164. Demographic, anamnestic, and therapeutical details are summarized in Table 1. Despite a higher presence of men in our population (116/164, 70.7—*p* < 0.001), we did not observe clinically relevant differences by gender. People older than 55 years represented about two-thirds of the overall population (106/164), and this reflects higher prevalence of comorbidities (*p* = 0.002), heavier polypharmacy (*p* = 0.015), and longer exposure to HIV-1 infection (*p* < 0.001).

### 3.2. Virologic Efficacy

**Figure 1:**
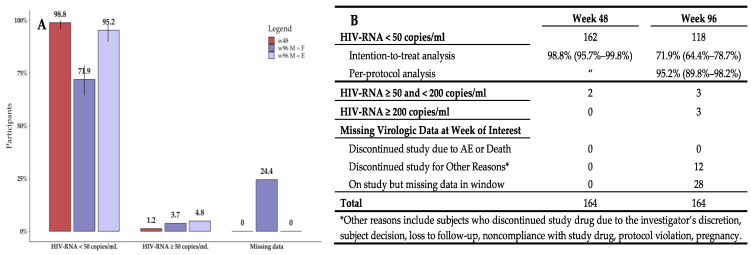
(**A**) Virologic control (HIV-RNA < 50 copies/mL), virologic failure (HIV-RNA ≥ 50 copies /mL) and missing virologic data at week 48 (w48) and week 96 (w96). M = F: missing equal failure—intention-to-treat analysis; M = E: missing equal excluded—per-protocol analysis. No missing data were recorded at w48, thus the M = E and the M = F analyses are equal (red bar). At w96 missing data were recorded; thus, the figure shows bars for both the intention-to-treat analysis considering missing as failure (dark violet), and the per-protocol analysis treating missing as excluded (pale violet). (**B**) Virologic outcome at w48 and w96 according to FDA guidelines [14].

Virologic results were evaluated according to the FDA snapshot algorithm, using HIV-RNA data available at the visits of interest [14]: Percentages of virologic response and failure, as well as percentages of missing virologic data for both BL, w48, and w96 are shown in Figure 1.

The rate of virologic control at w48 was 98.8% (162/164, CIs: 95.7–99.8%). The two participants with HIV-RNA > 50 copies/mL showed HIV-RNA values < 200 copies/mL, thus, they were maintained on BIC/FTC/TAF according to EACS guidelines [17] and were kept in follow-up at w96. One returned to virologic suppression at w96. The other, with detectable viral load at BL (HIV-RNA 128 copies/mL), experienced low-level viremia at w48 (HIV-RNA 77 copies/mL) and target undetectable at w96. No participant had missing virologic data at w48 nor underwent treatment interruption for any reason. Thus, the results of the intention-to-treat and the per-protocol analyses were equal.

At w96, virologic data were missing for a total of 40 participants. Out of these, 12 participants discontinued the study after moving to other clinical centers because of logistic or personal issues, whereas 28 were real missing data. Out of these twenty-eight, 17 participants were recently enrolled and thus they were not able to complete the w96 follow-up at the time of data collection, while 11 participants missed their appointment for the w96 follow-up visit. In the intention-to-treat analysis, a rate of virologic suppression at w96 of 71.9% (118/164, CIs: 64.4–78.7%) was reported. Nonetheless, in the per-protocol analysis, the rate of virologic suppression was 95.2% (118/124, CIs: 89.8–98.2%). At w96, six participants experienced virologic failure showing HIV-RNA > 50 copies/mL. Out of them, three participants had viral load <200 copies/mL. Before the switch to BIC/FTC/TAF, four of these were receiving a protease inhibitor-based ART regimen (darunavir), and the other two were on INSTI-based ART (one with dolutegravir and one with boosted elvitegravir). Participants showing virologic failure underwent a medical interview, and a blood sample was collected to perform a GRT. All six participants declared self-discontinuation of study drug due to logistic issues related to the access to therapy. However, all of them returned to <37 copies/mL after the adherence issues were resolved. No treatment-emergent RAMs were detected at the GRT.

**Figure 2:**
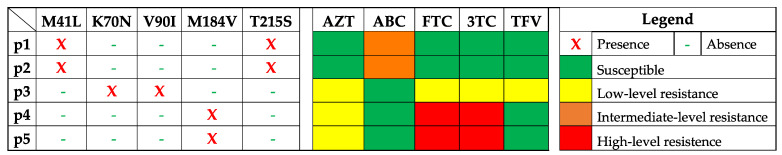
Genotypic profile of the 5 participants with RAMs at BL. 3TC: lamivudine; ABC: abacavir; AZT: zidovudine; BL: baseline; FTC: emtricitabine; p: participant; RAMs: resistance associated mutations; TFV: tenofovir.

At BL, 2 participants had M184V mutation and three had at least one thymidine analog mutation, thus harboring at least one RAM associated with lower susceptibility to the nucleos(t)ides in the BIC/FTC/TAF regimen. None of these showed virologic failure at both w48 and w96, and all completed the follow-up (Figure 2).

### 3.3. Immunologic Profile

#### 3.3.1. Overall BICTEL Cohort

Variations from BL to w48, characterized by a significant increase (*p* < 0.001) in CD4^+^ and CD8^+^ T cell counts and in CD4^+^/CD8^+^ ratio, were previously reported with a smaller sample size [13]. The same analysis on the current larger sample size (164) showed comparable results (Table 2). Comparing BL and w96 data, the median CD4^+^ T cell count improved from 580 (450–750) cells/μL to 790 (570–980) cells/μL (*p* < 0.001), with an absolute increase of 136 (−1–320) cells/μL and an MRD_(w96−BL)/BL_ of 24.2% (−0.2–50.4%). We did not observe any significant change in median CD8^+^ T cell count from BL to w96 (*p* = 0.861), nor of peripheral T lymphocytes between w48 and w96.

**Figure 3:**
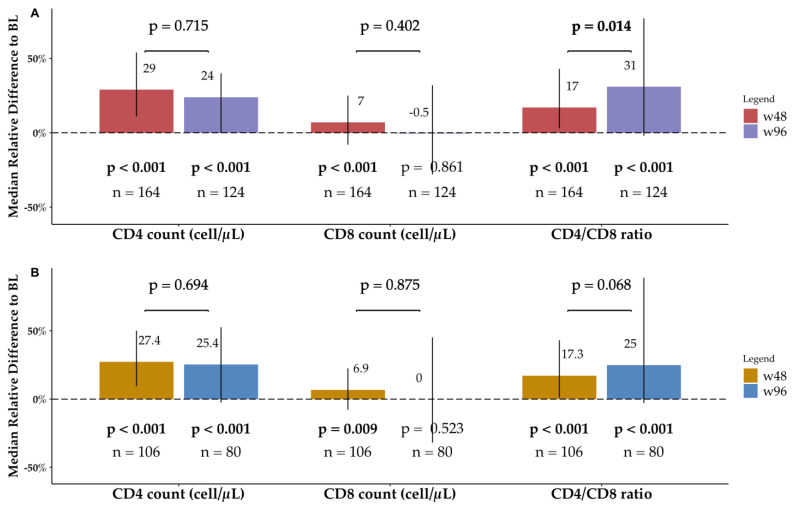
Immune profile changes over time. The bars show the median relative difference from baseline (BL) for the considered immune parameters at both week 48 (w48) and week 96 (w96). The *p*-value shown under each bar comes from the paired Wilcoxon test between BL and the considered time-point; the *p*-value above-between the w48-bar and the w96-bar comes from the paired Wilcoxon test between w48 and w96. Vertical lines show the interquartile range. (**A**) Data from the overall BICTEL cohort. (**B**) Data only from PLWH older than 55 years.

The significant improvement in the median CD4^+^/CD8^+^ ratio observed from 0.7 (0.6–0.9) at BL to 0.9 (0.8–1) (*p* < 0.001) at w48 was confirmed at w96 to 1.0 (0.7–1.4) (*p* < 0.001), rising by 0.19 (−0.02–0.6) from BL with an MRD_(w96−BL)/BL_ of 31.4% (−2–77.4%). Remarkably, median CD4^+^/CD8^+^ ratio at w96 was significantly higher than w48 values (*p* = 0.014), with a slight median absolute change from w48 of 0 (−0.2–0.4) and an MRD_(w96−w48)/w48_ of 2.2% (−17.3–40.9%) (Figure 3).

#### 3.3.2. PLWH Older than 55

Comparable results were detected among the elderly. We confirmed our previously reported results from w48 [13], showing a significant increase from BL to w96 in CD4^+^ T cell count (*p* < 0.001) and CD4^+^/CD8^+^ ratio (*p* < 0.001), but not in CD8^+^ T cell count (*p* = 0.523). No significant changes were detected between w48 and w96, including CD4^+^/CD8^+^ ratio (*p* > 0.05) (Table 2).

#### 3.3.3. Immune Changes among Time-Points as Function of BL Immune Status

To assess whether the observed immunological improvements were associated with BL immune status and to investigate the progressive positive trend in the CD4^+^/CD8^+^ ratio, we modeled in linear regression the immune changes over time, expressed as percentual MRD of the immune cell subsets between time-points, as a function of the BL CD4^+^ T cell count (BL CD4^+^ T cell count). Interestingly, both the w96 versus BL and the w48 versus BL comparisons showed a negative relationship of the CD4^+^ T cells MRD with the BL CD4^+^ T cell count (MRD_(w48−BL)/BL_: *p* < 0.001, MRD_(w96−BL)/BL_: *p* = 0.004, respectively), whilst the MRD_(w96−w48)/w48_ seemed not associated with basal immune status. Similar results were found among PLWH over 55 years.

**Figure 4:**
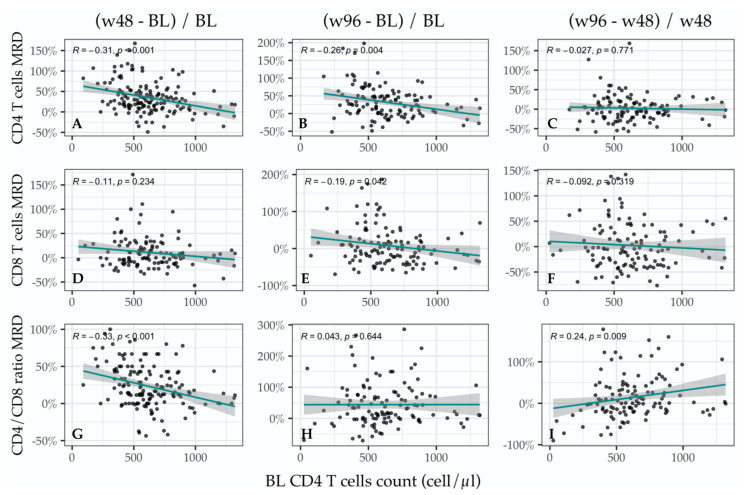
Immune changes among time points as function of BL immune status. The figure visualizes the results of our linear regression model assessing the immune changes between time-points as function of the baseline (BL) immune status, [formula: median relative difference (MRD) = a + b*(BL CD4^+^ T cell count)]. The left column (**A**,**D**,**G**) shows the comparisons between BL and week 48 (w48); the middle column (**B**,**E**,**H**) shows the comparisons between week 96 (w96) and BL; the right column (**C**,**F**,**I**) shows the comparisons between w96 and w48. The upper row is relative to CD4^+^ T cells count; the middle row to CD8^+^ T cells count; the lower row is relative to CD4^+^/CD8^+^ ratio. The significant associations shown in (**A**,**B**,**G**,**I**) were confirmed at the multivariate analysis.

With regard to the CD8^+^ T cell count, we were able to detect a slightly significant negative correlation solely between the MRD_(w96-BL)/BL_ and BL CD4^+^ T cell count (*p* = 0.042), but other time-point comparisons did not show any significant association, thus it could be due to chance. Stratifying by age, we saw that the significance was mainly driven by PLWH younger than 55. Surprisingly, we observed a changing behavior over time of the association between the CD4^+^/CD8^+^ ratio and BL CD4^+^ T cell count. Indeed, CD4^+^/CD8^+^ ratio change from BL negatively correlated with BL CD4^+^ T cell count when comparing w48 with BL (MRD_(w48−BL)/BL_: *p* < 0.001) but not when comparing w96 with BL (MRD_(w96−BL)/BL_: *p* = 0.644). Moreover, the w96 versus w48 comparison showed an opposite trend with a positive association between the CD4^+^/CD8^+^ ratio MRD_(w96−w48)/w48_ and the BL CD4^+^ T cell count (*p* = 0.009) (Figure 4).

To confirm such observations, we ran a multivariable regression model expressing the immune changes over time as a function of BL CD4^+^ T cell count and history of AIDS, time from HIV diagnosis, and time with HIV-RNA < 50 copies/mL before the switch. The multivariable analyses confirmed BL CD4^+^ T cell count as the unique independent predictor of the immune changes over time for CD4^+^ T cells and CD4^+^/CD8^+^ ratio (Supplementary Table S1).

### 3.4. Metabolic Profile

The current analysis largely confirmed our previous observations [13] regarding the w48 versus BL comparison. Briefly, we described the positive impact of BIC/FTC/TAF on the lipidic profile, characterized by a decline in TC (*p* < 0.001), LDL (*p* = 0.034), and TC/HDL ratio (*p* < 0.001) and a rise in HDL (*p* = 0.008), a statistically significant influence on weight gain (body weight: *p* = 0.006, BMI: *p* = 0.024) and renal function (creatinine: *p* = 0.034, eGFR: *p* < 0.001), and a neutral hepatic profile (AST: *p* = 0.189, ALT: *p* = 0.073). Median values and changes from BL are shown in Table 2. To further examine the different behavior of the serum lipidic markers over time, and in order to verify whether such changes were related to the pre-switch ART regimen, we attempted several stratification strategies. Although the pre-switch ART regimen groups were unbalanced, we were able to detect a significant change between BL and w48 in the TC/HDL ratio among all groups but NNRTI.

#### 3.4.1. Lipidic Profile, Body Weight, and BMI

In contrast to the results from the w48 versus BL comparison, no significant changes in lipidic profile, body weight, and BMI were observed between w96 and both BL and w48. The elderly showed similar results.

#### 3.4.2. Renal Function

We detected a significant increase in median serum creatinine levels from 0.94 (0.85–1.1) mg/dL at BL to 1.0 (0.87–1.2) mg/dL at w96 (*p* < 0.001), which corresponds to an MRD_(w96−BL)/BL_ of 4.2% (−3.7–15.2%). Alongside, we reported a reduction of the eGFR from 85 (73–97) mL/min/1.73 m^2^ at BL to 78 (65–92) mL/min/1.73 m^2^ (*p* = 0.002), corresponding to an MRD_(w96−BL)/BL_ of −5.5% (−14.5–3.6%). Again, excluding PLWH under 55 years from the analysis, we still detected the same trends among the elderly, with a significant rise in median serum creatinine levels from 0.9 (0.82–1) mg/dL at BL to 1.0 (0.88–1.22) mg/dL at w96 (*p* = 0.001), corresponding to an MRD_(w96−BL)/BL_ of 4.1% (−2.9–16.7%), as well as a parallel decline of the eGFR from 83 (71–94) mL/min/1.73 m^2^ at BL to 72 (63–88) mL/min/1.73 m^2^ (*p* = 0.016), corresponding to an MRD_(w96−BL)/BL_ of −6.2% (−17.4–4.1%). No significant changes were detected among w48 and w96 with regard to renal function, neither among the elderly nor overall.

#### 3.4.3. Hepatic Function

**Figure 5:**
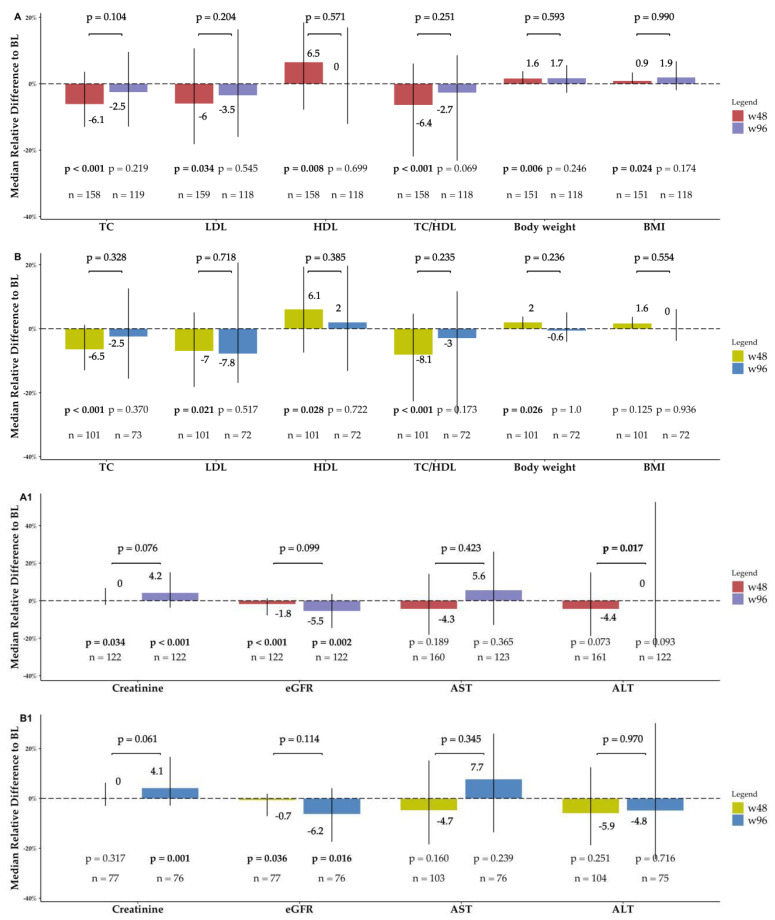
Metabolic changes over time. The bars show the median relative difference from baseline (BL) for the metabolic parameters considered at both week 48 (w48) and week 96 (w96). The *p*-value shown under each bar comes from the paired Wilcoxon test between BL and the considered time-point. The *p*-value above between the w48-bar and the w96-bar comes from the paired Wilcoxon test between w48 and w96. Vertical lines show the interquartile range. BMI: Body mass index; eGFR: Estimated glomerular filtrate rate, measured by the Chronic Kidney Disease Epidemiology Collaboration equation as mL/min/1.73 m^2^; TC: Total cholesterol; TC/HDL: Total cholesterol/LDL cholesterol ratio. (**A**,**A1**) Data from the overall BICTEL cohort. (**B**,**B1**) Data only from PLWH older than 55 years.

Liver function did not change from BL to w96, both in the overall cohort and in the sub-cohort of people older than 55. The w96 versus w48 comparison showed a not clinically, yet statistically significant rise in ALT and AST among the overall cohort (*p* = 0.017) and among PLWH younger than 55 (*p* = 0.011), respectively. Nonetheless, PLWH older than 55 did not show any hepatic functional change when observed alone (Figure 5).

### 3.5. Safety and Tolerability

No treatment-related serious AEs attributed to the use of BIC/FTC/TAF were reported in the medical records during the follow-up. No treatment discontinuation due to tolerability issues was reported.

## 4. Discussion

The current study brings some interesting evidence on the efficacy, safety, and tolerability of the once-daily single-tablet antiretroviral regimen BIC/FTC/TAF. To date, it provides a real-life confirmation of the virologic efficacy at w96, offering evidence of immune reconstitution and metabolic safety.

BIC/FTC/TAF showed high rates of virologic control in the per-protocol analysis at both w48 and w96. PLWH older than 55 presented comparable results with the overall cohort. Interestingly, our findings among the elderly are quite similar to previous reports, providing equal rates of virologic efficacy at w96 among PLWH older than 65 who underwent a therapeutic switch to BIC/FTC/TAF [18]. Indeed, in the overall analysis, 12/164 subjects (7.3%) underwent discontinuation of BIC/FTC/TAF, and none of them ascribed to virologic failure. Moreover, the six participants who experienced a virologic failure due to voluntary ART interruption returned to virologic control after improving their adherence to therapy. Finally, it is noteworthy that all the participants harboring at least one treatment resistance mutation associated with a lower susceptibility of at least one antiretroviral contained in this single-tablet regimen showed virologic control at w96. The two participants with M184V showed HIV-RNA < 50 copies/mL at w96. This is in line with the analysis conducted by Andreatta et al. on pre-existing RAM prevalence and impact on virologic outcome in the two pivot BIC/FTC/TAF switch studies 1878 and 1844, concluding that BIC/FTC/TAF is an effective treatment option for PLWH with virologic control, including those with evidence of archived NRTI RAMs [19]. Moreover, a recent retrospective review investigating the safety and efficacy of switching to BIC/FTC/TAF under virological control pooled 2034 participants from six clinical trials and found a prevalence of M184V of about 10%, with no relevant evidence of impairment of the virologic efficacy [20].

Our results highlight the magnitude of two pharmacological determinants of this INSTI-based single-tablet regimen: The high genetic barrier to resistance of bictegravir, as well as its long terminal half-life of elimination, the time required to divide the plasma concentration by two after reaching pseudo-equilibrium. Such pharmacological properties confer to the regimen powerful virologic efficacy, even in the presence of treatment RAMs to the nucleos(t)idic backbone, and meanwhile, grant an enduring inhibitory effect on viral replication, even in case of missed doses, especially among those PLWH more prone to self-managed ART interruptions [21,22,23,24].

An immune reconstitution is detectable in the BICTEL cohort. Our current results from 96 weeks of observation confirmed the positive effect of BIC/FTC/TAF on CD4^+^ T cell count and CD4^+^/CD8^+^ ratio previously reported at w48 [13]. The CD4^+^/CD8^+^ ratio at w96 showed a significant positive trend when compared with both BL and w48, suggesting a persistent improvement over time. Similar results among PLWH older than 55 were described for the CD4^+^ T cell count but not for the CD4^+^/CD8^+^ ratio. In this regard, our linear regression models provided deeper insight into the immune changes observed after the switch to BIC/FTC/TAF and their association with the BL immune status before the switch. Indeed, at w48 the immune changes from BL observed in CD4^+^ T cell count and CD4^+^/CD8^+^ ratio were inversely associated with the BL immune status, meaning that the lower BL CD4^+^ T cell count before the switch, the higher increase in CD4^+^ T cell count and CD4^+^/CD8^+^ ratio after the switch at w48.

However, we observed a lower association for the changes of CD4^+^ T cell count from BL at w96. Such results uncover an interesting relationship between the observed promising effect of BIC/FTC/TAF on immune reconstitution [13,25,26,27] and the degree of immune impairment at the time of the switch, even among participants with virologic control under ART. Interestingly, when we assessed the influence of the BL immune status before the switch on the peripheral immunological changes in a time period further from BL, like in the w96 versus w48 comparison, we did not see any detectable influence of BL immune status on both the CD4^+^ and the CD8^+^ T cell count changes. Other unknown factors could be the leading influencers of the immune system fluctuations the further PLWH get away from the switch. Nonetheless, we were able to appreciate a positive correlation between the CD4^+^/CD8^+^ ratio change between w96 and w48 and the BL CD4^+^ T cell count. Such shift over time from negative to positive in the correlation between the CD4^+^/CD8^+^ ratio changes and the BL immune status could be ascribed to major responsiveness to BIC/FTC/TAF in terms of immune reconstitution among those PLWH who are more immune-compromised by the disease and are responsible for the negative correlation observed immediately after the switch. After time from the switch, other influencing factors could overcome the quantitative immune restoration observed, thus accounting for the increase in CD8^+^ T cell count, especially among those more severely immunocompromised. Compared to them, PLWH with mild-low immune deficiency, who seemed to show lower improvement in immune markers just after the switch, demonstrated a slow yet persistent rise in CD4^+^/CD8^+^ ratio over time, probably due to higher resiliency and lower susceptibility to stressors associated with better immune conditions. Immune functional studies with cytokine profiles, alongside serum and intracellular pharmacokinetic assessment, are needed for a clearer understanding of the immune modulatory effect of BIC/FTC/TAF.

Since our cohort mostly includes PLWH older 55, our results add a remarkable piece of evidence to the inflammaging and frailty landscapes, which is currently coming up with a growing body of literature [28]. The ultimate clinical status of PLWH, especially in the elderly, relies on the chronic inflammation/immune reconstitution balance, impacting health span and quality of life. From this point of view, the metabolic profile also plays a pivotal role. In a recent meta-analysis, TAF-based backbone has been associated with weight gain, especially when combined with dolutegravir, with no relevant differences to BIC [29]. In our cohort, both lipidic and hepatic profiles seemed not affected by the ART switch when assessed at w96. In this regard, we are still detecting a significant improvement in serum fasting lipid parameters between the BL and w48, which is probably accounted for by the large prevalence (76%) of people coming from a boosted ART, PI-based in 60% of the cases. However, about 60% of participants were taking daily TDF before starting BIC/FTC/TAF, and this factor could have mitigated the expected advantage of a proactive or reactive switch to an un-boosted regimen because of the loss of the TDF-related statin-like effect. Considering that 30% of the switches were from a TAF-based backbone and about 25% from an INSTI-based regimen, we side with a recent review on the INSTI effect on lipidic profile, which concludes that, in the switch context, BIC has a superior lipid profile compared with boosted-PI, elvitegravir/cobicistat, and efavirenz [30]. Otherwise, the weight gain phenomenon, which is mostly observed among Black, Hispanic, and female populations (which are underrepresented in our study), could not apply to older PLWH, as suggested by two recent works reporting no weight gain among PLWH older than 65 years when switching to an INSTI-based regimen [18,31]. Indeed, overall participants’ lipidic profile, body weight, and BMI remained stable over time to w96, and stayed within the healthy range.

The renal function seemed to be negatively influenced by the switch to BIC/FTC/TAF after 96 weeks of observation. Our BL versus w48 analysis had already shown such a worsening trend, which was confirmed at w96, but with no significant difference between w48 and w96. The lack of evidence of a persisting negative influence on serum creatinine and eGFR between w48 and w96 leads us to indicate the large prevalence of boosted PI and TDF among the pre-switch ART regimens as responsible for the worsening in renal function observed. Longer studies, coupling serum follow-up with longitudinal urine analytes monitoring, are needed to assess whether the tubular TDF- and/or cobicistat-related tubulopathy could have influenced renal function. Nonetheless, median eGFR remained within the same rank (grade 2, 60–89 mL/min/1.73 m^2^) without any deterioration to lower grades.

The study has some limitations. First, the retrospective design of the study limits the accuracy of the analysis on the impact of the switch since a prospective design would be more appropriate. Second, the cohort does not have a comparator of people who did not switch to BIC/FTC/TAF, nor of PLWH switching to other regimens, and hence the described changes cannot be attributed solely to the switch. Third, not all the participants enrolled at BL have completed the follow-up, thus lowering the statistical power of the analyses involving w96. Fourth, we computed the eGFR using the old CKD-EPI formula when the usage of the new formula released in 2021 is warranted.

## 5. Conclusions

After 96 weeks of observations, BIC/FTC/TAF is revealed as an effective, safe, and well-tolerated strategy for PLWH, regardless of age and sex. The virologic efficacy of BIC/FTC/TAF depends on its high genetic barrier allowing virologic control even in the presence of RAMs to NRTI, such as M184V. The promising immune reconstitution observed in the BICTEL cohort warrants further examinations of the immune modulatory effects of BIC/FTC/TAF among PLWH with diverse severity of immune deficit. A neutral metabolic profile makes BIC/FTC/TAF a favorable choice among all PLWH, including those with older age, multiple comorbidities, high pill burden, and sub-optimal adherence to ART. In this regard and to confirm such observations, it is our intention to follow up on the participants to week 144.

## Figures and Tables

**Table 1:** Demographics of the overall population, and by age older than 55 years and by sex. *p*-values < 0.05 are highlighted in bold. ABC: abacavir; AIDS: adquired immune deficency syndrome; ART: antiretroviral therapy; HBV: hepatitis B virus; HCV: hepatitis C virus; INSTI: integrase strand transfer inhibitors; NNRTI: non-nucleosidic reverse transcriptase inhibitors; PI: protease inhibitors; TDF: tenofovir disoproxil fumarate; TAF: tenofovir alafenamide fumarate.

	Overall	Over 55 Years	Under 55 Years	*p*	Female	Male	*p*
	*n* = 164	*n* = 106	*n* = 58		*n* = 48	*n* = 116	
	m/# (IQR/%)	m/# (IQR/%)	m/# (IQR/%)		m/# (IQR/%)	m/# (IQR/%)	
**Sex**							
Female	48 (29.3%)	27 (25.5%)	21 (36.2%)	0.156	48 (100%)	0 (0%)	**<0.001**
Male	116 (70.7%)	79 (74.5%)	37 (63.8%)		0 (0%)	116 (100%)	
**Age** (years)	57 (47–61)	59 (57–64)	41 (37–48)	**<0.001**	56 (46–58)	57 (47–62)	0.0893
**Smoking** (yes)	108 (65.9%)	68 (64.2%)	40 (69.0%)	0.607	28 (58.3%)	80 (69.0%)	0.209
**Time from HIV-1 diagnosis** (years)	16 (9.5–22)	19 (12–24)	12 (6.0–15)	**<0.001**	19 (10–24)	15 (8.0–21)	0.113
**History of AIDS diagnosis** (yes)	55 (33.5%)	40 (37.7%)	15 (25.9%)	0.166	15 (31.3%)	40 (34.5%)	0.72
**HBV co-infection** (yes)	12 (7.3%)	8 (7.5%)	4 (6.9%)	1	2 (4.2%)	10 (8.6%)	0.512
**Former HCV infection** (yes)	18 (11.0%)	15 (14.2%)	3 (5.2%)	0.116	8 (16.7%)	10 (8.6%)	0.17
**HIV-1-related non-AIDS comorbidities** (≥1)	98 (59.8%)	73 (68.9%)	25 (43.1%)	**0.002**	30 (62.5%)	68 (58.6%)	0.727
How many	1 (0–1)	1 (0–2.0)	0 (0–1)	**<0.001**	1 (0–1)	1 (0–1)	0.591
Osteoporosis	27 (16.5%)	23 (21.7%)	4 (6.9%)	**0.015**	8 (16.7%)	19 (16.4%)	1
Type 2 Diabetes	12 (7.3%)	10 (9.4%)	2 (3.4%)	0.217	4 (8.3%)	8 (6.9%)	0.748
Hypertension	32 (19.5%)	26 (24.5%)	6 (10.3%)	**0.038**	8 (16.7%)	24 (20.7%)	0.667
Major Cardiovascular events	12 (7.3%)	12 (11.3%)	0 (0%)	**0.008**	1 (2.1%)	11 (9.5%)	0.183
**Other than ART co-medications**							
≥1	69 (42.1%)	49 (46.2%)	20 (34.5%)	0.186	26 (54.2%)	43 (37.1%)	0.056
≥2	41 (25.0%)	33 (31.1%)	8 (13.8%)	**0.015**	12 (25.0%)	29 (25.0%)	1
**Pre-switch ART regimen**							
INSTI	38 (23.2%)	23 (21.7%)	15 (25.9%)		9 (18.8%)	29 (25.0%)	
NNRTI	20 (12.2%)	11 (10.4%)	9 (15.5%)	0.545	3 (6.3%)	17 (14.7%)	0.134
PI	99 (60.4%)	68 (64.2%)	31 (53.4%)		32 (66.7%)	67 (57.8%)	
PI+INSTI	7 (4.3%)	4 (3.8%)	3 (5.2%)		4 (8.3%)	3 (2.6%)	
TDF-based backbone	95 (57.9%)	67 (63.2%)	28 (48.3%)	0.071	22 (45.8%)	73 (62.9%)	0.056
TAF-based backbone	49 (29.9%)	26 (24.5%)	23 (39.7%)	0.051	15 (31.3%)	34 (29.3%)	0.852
ABC-based backbone	5 (3.0%)	3 (2.8%)	2 (3.4%)	1	4 (8.3%)	1 (.9%)	0.026
Boosted-regimen	124 (75.6%)	79 (74.5%)	45 (77.6%)	0.708	40 (83.3%)	84 (72.4%)	**0.165**
Dual therapy	10 (6.1%)	8 (7.5%)	2 (3.4%)	0.497	4 (8.3%)	6 (5.2%)	0.481
**Reason to switch**							
Adherence	4 (2.4%)	3 (2.8%)	1 (1.7%)	0.536	3 (6.3%)	1 (0.9%)	0.279
Adverse events	1 (0.6%)	1 (0.9%)	0 (0%)		0 (0%)	1 (0.9%)	
Proactive	15 (9.1%)	7 (6.6%)	8 (13.8%)		5 (10.4%)	10 (8.6%)	
Simplification	110 (67.1%)	71 (67.0%)	39 (67.2%)		32 (66.7%)	78 (67.2%)	
Toxicity	34 (20.7%)	24 (22.6%)	10 (17.2%)		8 (16.7%)	26 (22.4%)	

**Table 2:** Immunological and metabolic changes between baseline, week 48 and week 96. The table summarizes raw values of and changes between baseline (BL), week 48 (w48) and week 96 (w96). On the left side the table reports the medians (first quartile–third quartile) values at the three time-points for the variables of interest. On the right side the table reports three columns, one for each comparison (w96 versus BL, w48 versus BL and w96 versus w48). For each comparison the table reports three columns, one for the absolute difference between time-points (e.g., w96–BL), another for the percentual median relative difference (MRD %) between time-points (e.g., 100 × [week 96 value—BL value]/BL value), and a third for the p-values coming from the paired Wilcoxon test between time-points (paired Wilcoxon test between w96 and BL). *p*-values < 0.05 are highlighted in bold. eGFR: estimated glomerular filtrate rate, measured by the Chronic Kidney Disease Epidemiology Collaboration equation as mL/min/1.73 m^2^; TC: Total cholesterol. **A**. Data from the overall BICTEL cohort. **B**. Data only from PLWH older than 55 years.

**A.** **Overall Cohort (n: 164)**	**BL**	**w48**	**w96**	**w96 vs. BL**			**w48 vs. BL**			**w96 vs. w48**		
				**Absolute Difference**	**MRD %**	***p***	**Absolute Difference**	**MRD %**	***p***	**Absolute Difference**	**MRD %**	***p***
**CD4^+^ T cell count (cell/μL)**	580 (450–750)	760 (590–1000)	790 (570–980)	136 (−1–320)	24.2 (−0.2–5.4)	**<0.001**	163 (67–281)	29 (11.1–53.9)	**<0.001**	–8 (−126–132)	−1.6 (−19–18)	0.715
**CD8^+^ T cell count (cell/μL)**	750 (580–1000)	850 (640–1000)	790 (550–1100)	−6 (−230–254)	−0.5 (−28.1–31.6)	0.861	56 (−58–183)	7.1 (−8.3–25.2)	**<0.001**	−35 (−290–209)	−5.2 (−32.8–29)	0.402
**CD4^+^/CD8^+^ ratio**	0.70 (0.60–0.90)	0.90 (0.80–1.0)	1.0 (0.72–1.4)	0.19 (−0.02–0.6)	31.4 (−2–77.4)	**<0.001**	0.14 (0.01–0.3)	17.3 (3.5–43.1)	**<0.001**	0 (−0.2–0.4)	2.2 (−17.3–4.9)	**0.014**
**Total Cholesterol (mg/dL)**	190 (170–210)	180 (160–200)	180 (160–210)	−4 (−25–16)	−2.5 (−12.9–9.6)	0.219	−12 (−24–6)	−6.1 (−13–3.6)	**<0.001**	3.5 (−15–24)	2.2 (−7.2–14.1)	0.104
**LDL (mg/dL)**	110 (87–130)	100 (83–130)	110 (88–140)	−3 (−17–19)	−3.5 (−16–16.4)	0.545	−6 (−17–12)	−6 (−18.2–1.7)	**0.034**	2.9 (−16.2 – 21)	3.5 (−14–24.4)	0.204
**HDL (mg/dL)**	50 (41–58)	52 (44–62)	53 (45–63)	0 (−7–7)	0 (−12.1–17)	0.699	3 (−5–8)	6.5 (−7.8–18.5)	**0.008**	0 (−9–7)	0 (−15.8–15.1)	0.571
**TC/HDL ratio**	3.7 (3.0–4.7)	3.4 (2.8–4.1)	3.6 (3.0–4.5)	−0.12 (−0.78–0.29)	−2.7 (−23.2–8.6)	0.069	−0.22 (−0.88–0.18)	−6.4 (−21.9–6.1)	**<0.001**	0.1 (−0.5–0.7)	2 (−14.5–22.2)	0.251
**Body Weight (Kg)**	77 (70–84)	78 (71–85)	78 (65–84)	1.3 (−2.5–4)	1.7 (−2.7–5.6)	0.246	1 (0–3)	1.6 (0–3.8)	**0.006**	0 (−5–4)	0 (−5.8–4.6)	0.593
**BMI (Kg/m^2^)**	25 (23–28)	26 (23–29)	25 (23–27)	0.55 (−0.43–1.67)	1.9 (−1.9–6.8)	0.174	0.28 (0–0.88)	0.9 (0–3.4)	**0.024**	0.3 (−1.6–1.4)	1.1 (−5.1–5.4)	0.990
**Creatinine (mg/dL)**	0.94 (0.85–1.1)	1.0 (0.84–1.1)	1.0 (0.87–1.2)	0.05 (−0.03–0.14)	4.2 (−3.7–15.2)	**<0.001**	0 (−0.2–0.08)	0 (−2.2–6.8)	**0.034**	0 (−0.1–0.1)	1.3 (−5.5–12.9)	0.076
**eGFR**	85 (73–97)	80 (70–93)	78 (65–92)	−4.15 (−11.42–3.27)	−5.5 (−14.5–3.6)	**0.002**	−1.6 (−6.35–1)	−1.8 (−7.8–1.3)	**<0.001**	–1.8 (−8.1–5.7)	−2.1 (−9.5–7.6)	0.099
**AST (mg/dL)**	21 (17–24)	20 (17–23)	21 (18–26)	1 (−2.5–5)	5.6 (−12.9–26.1)	0.365	−1 (−4–2)	−4.3 (−18.2–14.3)	0.189	1 (−2–5)	4 (−10–27.8)	0.423
**ALT (mg/dL)**	21 (16–26)	20 (16–25)	20 (15–32)	0 (−6–9)	0 (−24.8–52.6)	0.093	−1 (−4–3)	−4.4 (−18.8–15.1)	0.073	0 (−5–7)	0 (19.6–36.9)	**0.017**
**B.** **PLWH Older than 55 (n: 106)**	**BL**	**w48**	**w96**	**w96 vs. BL**			**w48 vs. BL**			**w96 vs. w48**		
				**Absolute Difference**	**MRD %**	***p***	**Absolute Difference**	**MRD %**	***p***	**Absolute Difference**	**MRD %**	***p***
**CD4^+^ T cell count (cell/μL)**	580 (460–730)	760 (550–960)	770 (550–920)	155 (11–312)	25.4 (2.5–52.6)	**<0.001**	160 (53–264)	27.4 (9.5–5.1)	**<0.001**	15 (−92–119)	2.7 (−13.2–18.3)	0.694
**CD8^+^ T cell count (cell/μL)**	750 (580–1000)	850 (660–1000)	800 (590–1100)	0 (−248–370)	0 (−31.9–45.2)	0.523	50 (−54–141)	6.9 (−7.8–22.6)	**0.009**	–19 (−273–291)	−3.3 (−34.9–41.4)	0.875
**CD4^+^/CD8^+^ ratio**	0.70 (0.60–0.82)	0.90 (0.80–1.0)	0.92 (0.62–1.4)	0.13 (−0.02–0.6)	25 (−2.9–88.9)	**<0.001**	0.13 (0.01–0.3)	17.3 (1.1–43.1)	**<0.001**	0 (−0.2–0.4)	5.5 (−19.9–40)	0.068
**Total Cholesterol (mg/dL)**	190 (170–210)	180 (160–210)	190 (160–220)	−6 (−29–21)	−2.5 (−15.6–12.6)	0.370	−12 (−23–2)	−6.5 (−13–1.2)	**<0.001**	2 (−16–24)	0.7 (−7.3–14.3)	0.328
**LDL (mg/dL)**	110 (88–140)	100 (84–140)	110 (81–140)	−11 (−20–22)	−7.8 (−16.9–2.7)	0.517	−7 (−17–6)	−7 (−18.2–5.1)	**0.021**	1 (−20–22)	0.7 (−2.3–27.9)	0.718
**HDL (mg/dL)**	47 (41–57)	50 (43–60)	52 (44–63)	1 (−7–7)	2 (−13.2–19.7)	0.722	3 (−4–8)	6.1 (−7.5–19.4)	**0.028**	–1 (−10–8)	−1.6 (−19.3–17)	0.385
**TC/HDL ratio**	3.9 (3.4–4.7)	3.6 (2.9–4.3)	3.6 (3.1–4.6)	−0.13 (−0.82–0.43)	−3 (−26.7–11.7)	0.173	−0.31 (−0.97–0.16)	−8.1 (−22.7–4.7)	**<0.001**	0.2 (−0.5–0.7)	5.3 (−15.5–22.8)	0.235
**Body Weight (Kg)**	78 (71–88)	80 (71–89)	79 (65–85)	−0.5 (−3–4)	−0.6 (−4.1–5.1)	1.000	1.5 (0–3)	2 (0–3.8)	**0.026**	−2 (−5–4)	−2.2 (−7.8–5.1)	0.236
**BMI (Kg/m^2^)**	26 (24–29)	27 (25–29)	25 (24–28)	0 (−1.01–1.55)	0 (−3.8–6.1)	0.936	0.4 (0–0.9)	1.6 (0–3.7)	0.125	−0.3 (−1.8–1.4)	−1.1 (−6.4–5.3)	0.554
**Creatinine (mg/dL)**	0.90 (0.82–1.0)	0.98 (0.81–1.1)	1.0 (0.88–1.2)	0.05 (−0.03–0.15)	4.1 (−2.9–16.7)	**0.001**	0 (−0.03–0.07)	0 (−3–6.3)	0.317	0 (0–1)	1.3 (−3.7–16)	0.061
**eGFR**	83 (71–94)	78 (69–92)	72 (63–88)	−4.85 (−16.73–3.83)	−6.2 (−17.4–4.1)	**0.016**	−0.7 (−6.1–1.3)	−0.7 (−7.1–1.9)	**0.036**	−2.2 (−12–4.7)	−3.1 (−15.4–6.7)	0.114
**AST (mg/dL)**	21 (18–24)	20 (17–23)	21 (19–26)	1 (−3–5)	7.7 (−13.6–26)	0.239	−1 (−4–2)	−4.7 (−18.4–15.2)	0.160	0 (−2–4)	0 (−11.9–2.2)	0.345
**ALT (mg/dL)**	21 (17–26)	21 (16–25)	19 (15–31)	−1 (−5–7)	−4.8 (−24.4–3.2)	0.716	1 (−4–2)	−5.9 (−18.8–12.5)	0.251	–1 (−5–6)	−5.3 (−24.4–30)	0.970

## Data Availability

Data available on request. The data presented in this study are available on request from the corresponding author.

## Supplementary Materials

The following supporting information can be downloaded at: https://www.mdpi.com/article/10.3390/v15061222/s1.

## References

[1] Gilead Sciences (2018). Biktarvy^®^ (Bictegravir, Emtricitabine, and Tenofovir Alafenamide): US Prescribing Information. https://www.accessdata.fda.gov/.

[2] Gilead Sciences (2018). Biktarvy 50 mg/200 mg/25 mg Film-Coated Tab-Lets: EU Summary of Product Characteristics. http://www.371ema.europa.eu/.

[3] Markham A. (2018). Bictegravir: First Global Approval. Drugs.

[4] Tsiang M., Jones G.S., Goldsmith J., Mulato A., Hansen D., Kan E., Tsai L., Bam R.A., Stepan G., Stray K.M. (2016). Antiviral Activity of Bictegravir (GS-9883), a Novel Potent HIV-1 Integrase Strand Transfer Inhibitor with an Improved Resistance Profile. Antimicrob. Agents Chemother..

[5] Di Perri G. (2021). Tenofovir alafenamide (TAF) clinical pharmacology. Le Infez. Med..

[6] Daar E.S., DeJesus E., Ruane P., Crofoot G., Oguchi G., Creticos C., Rockstroh J.K., Molina J.-M., Koenig E., Liu Y.-P. (2018). Efficacy and safety of switching to fixed-dose bictegravir, emtricitabine, and tenofovir alafenamide from boosted protease inhibitor-based regimens in virologically suppressed adults with HIV-1: 48 week results of a randomised, open-label, multicentre, phase 3, non-inferiority trial. Lancet HIV.

[7] Kityo C., Hagins D., Koenig E., Avihingsanon A., Chetchotisakd P., Supparatpinyo K., Gankina N., Pokrovsky V., Vo-ronin E., Stephens J.L. Switching to bictegravir/emtricitabine/tenofovir alafenamide in women [poster no. 500]. Proceedings of the Conference on Retroviruses and Opportunistic Infections.

[8] Molina J.-M., Ward D., Brar I., Mills A., Stellbrink H.J., López-Cortés L., Ruane P., Podzamczer D., Brinson C., Custodio J. (2018). Switching to fixed-dose bictegravir, emtricitabine, and tenofovir alafenamide from dolutegravir plus abacavir and lamivudine in virologically suppressed adults with HIV-1: 48 week results of a randomised, double-380 blind, multicentre, active-controlled, phase 3, non-inferiority trial. Lancet HIV.

[9] Pepperrell T., Hill A., Moorhouse M., Clayden P., McCann K., Sokhela S., Serenata C., Venter W.D.F. (2020). Phase 3 trials of new antiretrovirals are not representative of the global HIV epidemic. J. Virus Erad..

[10] Marcus J.L., Leyden W.A., Alexeeff S.E., Anderson A.N., Hechter R.C., Hu H., Lam J.O., Towner W.J., Yuan Q., Horberg M.A. (2020). Comparison of Overall and Comorbidity-Free Life Expectancy Between Insured Adults With and Without HIV Infection, 2000–2016. JAMA Netw. Open.

[11] Smit M., Brinkman K., Geerlings S., Smit C., Thyagarajan K., van Sighem A., de Wolf F., Hallett T.B. (2015). Future challenges for clinical care of an ageing population infected with HIV: A modelling study. Lancet Infect. Dis..

[12] Horberg M.A., Hurley L.B., Klein D.B., Towner W.J., Kadlecik P., Antoniskis D., Mogyoros M., Brachman P.S., Remmers C.L., Gambatese R.C. (2015). The HIV care cascade measured over time and by age, sex, and race in a large national integrated care system. AIDS Patient Care STDS.

[13] Lazzaro A., Cacciola E.G., Borrazzo C., Innocenti G.P., Cavallari E.N., Mezzaroma I., Falciano M., Fimiani C., Mastroianni C.M., Ceccarelli G. (2021). Switching to a Bictegravir Single Tablet Regimen in Elderly People Living with HIV-1: Data Analysis from the BICTEL Cohort. Diagnostics.

[14] US Food and Drug Administration (2015). Human Immunodeficiencyvirus-1 Infection: Developing Antiretroviral Drugs for Treatment. https://www.fda.gov/regulatory-information/search-fda-guidance-documents/human-immunodeficiency-virus-1-infection-developing-antiretroviral-drugs-treatment.

[15] Levey A.S., Stevens L.A., Schmid C.H., Zhang Y.L., Castro A.F., Feldman H.I. (2009). CKD-EPI (Chronic Kidney Disease Epidemiology Collaboration). A new equation to estimate glomerular filtration rate. Ann. Intern. Med..

[16] Division of AIDS (DAIDS) Table for Grading the Severity of Adult and Pediatric Adverse Events. https://rsc.niaid.nih.gov/sites/default/files/daidsgradingcorrectedv21.pdf.

[17] European AIDS Clinical Society (2021). Guidelines Version 11.1. http://eacsociety.org.

[18] Maggiolo F., Rizzardini G., Molina J.M., Pulido F., De Wit S., Vandekerckhove L., Berenguer J., D’Antoni M.L., Blair C., Chuck S.K. (2023). Bictegravir/emtricitabine/tenofovir alafenamide in older individuals with HIV: Results of a 96-week, phase 3b, open-label, switch trial in virologically suppressed people ≥65 years of age. HIV Med..

[19] Andreatta K., Willkom M., Martin R., Chang S., Wei L., Liu H., Liu Y.-P., Graham H., Quirk E., Martin H. (2019). Switching to bictegravir/emtricitabine/tenofovir alafenamide maintained HIV-1 RNA suppression in participants with archived antiretroviral resistance including M184V/I. J. Antimicrob. Chemother..

[20] Sax P.E., Andreatta K., Molina J.-M., Daar E.S., Hagins D., Acosta R., D’antoni M.L., Chang S., Martin R., Liu H. (2022). High efficacy of switching to bictegravir/emtricitabine/tenofovir alafenamide in people with suppressed HIV and preexisting M184V/I. AIDS.

[21] Tang M.W., Shafer R.W. (2012). HIV-1 antiretroviral resistance: Scientific principles and clinical applications. Drugs.

[22] Toutain P.L., Bousquet-Mélou A. (2004). Plasma terminal half-life. J. Vet. Pharmacol. Ther..

[23] Parienti J.-J., Fournier A.L., Cotte L., Schneider M.-P., Etienne M., Unal G., Perré P., Dutheil J.-J., Morilland-Lecoq E., Chaillot F. (2021). Forgiveness of Dolutegravir-Based Triple Therapy Compared With Older Antiretroviral Regimens: A Prospective Multicenter Cohort of Adherence Patterns and HIV-RNA Replication. Open Forum Infect. Dis..

[24] Elliot E., Amara A., Jackson A., Moyle G., Else L., Khoo S., Back D., Owen A., Boffito M. (2016). Dolutegravir and elvitegravir plasma concentrations following cessation of drug intake. J. Antimicrob. Chemother..

[25] Benedetti S., Altobelli D., De Socio G., Lanzi A., Gamboni G., Francisci D. (2022). Real-life monocentric Biktarvy cohort from Perugia. HIV Glasgow 2022, Poster P129. J. Int. AIDS Soc..

[26] d’Arminio Monforte A., Tavelli A., Cingolani A., Taramasso L., Mussini C., Piconi S., Calcagno A., Orofino G., Cicalini S., Castagna A. Effectiveness of Bictegravir/Emtricitabine/Tenofovir Alafenamide (BIC/FTC/TAF) as Switch Strategy in Virologically Suppressed: Real-World Data from the ICONA Cohort. HIV Glasgow 2022, Poster P098. https://hivglasgow.org/wp-content/uploads/2023/01/P098_dArminio_Monforte.pdf.

[27] Trottier B., Antinori A., De Wet J., Duvivier C., Elinav H., Esser S., Ghosn J., den Hollander J., Lamber J., Milinkovic A. Bictegravir/emtricitabine/tenofovir alafenamide (B/F/TAF) for the treatment of people living with HIV: 24-month (24M) analyses by age, race, sex, adherence and late presentation in a multi-country cohort study. HIV Glasgow 2022, Poster P067. https://onlinelibrary.wiley.com/doi/10.1002/jia2.26009.

[28] Kehler D.S., Milic J., Guaraldi G., Fulop T., Falutz J. (2022). Frailty in older people living with HIV: Current status and clinical management. BMC Geriatr..

[29] Kanters S., Renaud F., Rangaraj A., Zhang K., Limbrick-Oldfield E., Hughes M., Ford N., Vitoria M. (2022). Evidence synthesis evaluating body weight gain among people treating HIV with antiretroviral therapy-a systematic literature review and network meta-analysis. EClinicalMedicine.

[30] Saumoy M., Sanchez-Quesada J.L., Ordoñez-Llanos J., Podzamczer D. (2021). Do All Integrase Strand Transfer Inhibitors Have the Same Lipid Profile? Review of Randomised Controlled Trials in Naïve and Switch Scenarios in HIV-Infected Patients. J. Clin. Med..

[31] Guaraldi G., Calza S., Milic J., Calcagno A., Focà E., Rota M., Renzetti S., Celotti A., Siano M., Celesia B.M. (2021). Dolutegravir is not associated with weight gain in antiretroviral therapy experienced geriatric patients living with HIV. AIDS.

